# Mind Sports: Exploring Motivation and Use of Cognitive Strategies in Bridge

**DOI:** 10.3390/ijerph20064968

**Published:** 2023-03-11

**Authors:** Liat Hen-Herbst, Liron Lamash, Yael Fogel, Sonya Meyer

**Affiliations:** 1 Department of Occupational Therapy, Faculty of Health Sciences, Ariel University, Ariel 4077603, Israel; 2 Department of Occupational Therapy, Faculty of Welfare and Health Sciences, University of Haifa, Haifa 3498838, Israel

**Keywords:** bridge, mind sport, mental exercise, cognitive strategies, intellectual skills

## Abstract

The game of Bridge is one of the world’s most widely played mind-sport games. A growing number of people consider it a meaningful leisure activity and are motivated to play. The aim of this study was to describe a sample of Bridge players in Israel in terms of demographics, player records, motivations, and cognitive strategies used to play Bridge and examine the associations between these variables. A sample of 488 Bridge players’ completed an online demographic questionnaire, the Bridge Motivational Factors Checklist, and the Bridge Cognitive Strategies Questionnaire. Most players in the study were men with a mean age of 68.7 years and a Bridge player level between Vice Master and Senior Master. Most players play bridge because “bridge is a fun game”. Strategy use that occurred in-game (versus pre- or postgame) obtained the highest mean score. Because Bridge is a strategy game that can be played from childhood to older adulthood, it is important to continue research to further understand the nature and different aspects of the cognitive strategy used among Bridge players and in other mind sports.

## 1. Introduction

Since 1941, 44% of Americans have played Bridge, a game “recommended as a means of learning social skills” [1]. As of 2006, Bridge became one of the world’s most widely played, stimulating, and challenging card games, which requires skill, concentration, and practice [2]. Four players typically play the game in casual environments or competitive tournaments [3]. It is a game of teamwork that involves building and maintaining relationships between partners. At the same time, each dyad develops a unique system, giving the partnerships distinct styles depending on with and against whom they are playing [4].

The International Olympic Committee, through the World Bridge Federation (WBF) founded in 1958 (formerly the International Bridge League of 1932), recognizes Bridge as a mind sport [5]. The term mind sport, used for over a decade [6], refers to a game based primarily on intellectual rather than physical skills [4]. The WBF website [7] stated, “The WBF has shown strong and steady growth, and its membership now comprises 116 National Bridge Organizations with approximately 1,000,000 affiliated members who participate actively in competitive bridge events (locally, nationally and internationally)”.

The Israel Bridge Association is a member of the Eilat Association, the umbrella organization of non-Olympic sports in Israel. There are approximately 8500 members of the Israel Bridge Federation and approximately 100,000 bridge players in Israel who are not federation members [8]. This article aims to characterize Bridge players in Israel, highlighting the motivation and cognitive strategies required to play Bridge.

Most available scientific literature is related to examining playing Bridge among people with neurodegenerative diseases such as dementia [9] and Alzheimer’s disease [10]. For example, Malysa [11] developed a program encouraging older adults to play Bridge to reduce the risk of Alzheimer’s disease and other dementias. In addition, recent studies involved elite Bridge players’ emotions and motivations concerning the game [12,13,14]. Although Bridge has developed over the years, it is still considered a game for the elderly [15]. The average age of English Bridge Union members was 55 years in 2006 [16] and 67 years for American Contract Bridge League members in 2005 [17]. The literature documented that a high percentage of Bridge players are men [1]. However, information on the age, gender, and health status characteristics of Bridge players in Israel is scarce.

Bridge players’ records include Master points awarded by Bridge organizations to individuals for success in competitive tournaments under their auspices. The Master points concept endeavors to indicate a player’s true value. The typical Israeli categories are Registered Player, Vice Master, Master, Senior Master, Life Master, and Grand Life Master. Most competitions award local Master points for above-average achievements according to the player’s level. National points are awarded at district and national events or special competitions, and international points are awarded at international events.

To date, the literature on the game of Bridge has focused mainly on elite [4] “edge sachets” or disease-impaired players and less on average Bridge players. The elite level includes top amateurs and professional players (full- or part-time) alongside high-profile sponsors.

Galbraith et al. [18] examined Bridge players’ responses to an open-ended question on why people play Bridge. Enjoying Bridge was prominent within all age categories; people reported that the game was enjoyable, addictive, or interesting. The main themes that emerged from their survey were that 51.6% of the participants played the game for enjoyment, 37.5% for the mental challenge, and 32% for fun, social interaction, or enjoyment of having company.

Over the past decade, researchers have explored the link between Bridge and players’ well-being [3,19]. Punch and colleagues [4] recently published results of qualitative research investigating motivations and participation in tournaments among 52 elite Bridge players from the United States and Europe. Their findings supported that Bridge is a central part of professional players’ lives; these players commit to and persevere in their role with more intense practice and play than amateurs. Their commitment to the pursuit included actively resisting professionalization, which they may view as incompatible with what they value from participating in Bridge as a leisure activity [4].

Players strongly indicated the mental challenge that Bridge presents. Although this generically could be referred to as a challenge, another theme frequently referred to Bridge as a mental exercise. People spoke of Bridge in terms of workouts, gymnastics, and exercises carried out mentally. This response is interesting because it implied that Bridge was a mental sport, usually a reason for playing. In other words, some people said they enjoyed how Bridge challenged (exercised) their brains and thinking [18].

Alongside intellect, endurance, technical and communication skills, and social interaction are central to Bridge [4]. To play well, one needs to use a variety of strategies. Bridge players display the capacity to make crucial gameplay decisions based on incomplete information while judging their partner’s card play and opponents’ impressions correctly or incorrectly. They need to control their frustration with their own or their partners’ failings so as not to give their opponents an advantage through a verbal outburst—while also changing game plans based on the ever-evolving card game [13]. To date, no study has mapped the potential strategies of Bridge players. Moreover, no study has analyzed interrelationships between gender differences, player records, motivations, and cognitive strategies in Bridge.

This study is the first among Bridge players in Israel. The study’s aims were to describe the characteristics of a sample of Bridge players in Israel in terms of demographics, player records, motivations, and strategies used to play Bridge and examine the associations between these variables.

## 2. Materials and Methods

### 2.1. Participants

In this cross-sectional study, inclusion criteria were women and men aged 18 years and older, registered with the Israeli Bridge Association, with a sufficient command of the Hebrew language to answer the online questionnaires using Qualtrics software, and were active bridge players who accumulated at least “local points” (i.e., participated in local bridge competitions organized by the Israeli Bridge association). The exclusion criteria were players younger than 18 years, nonactive Bridge players (i.e., had no registered points with the Bridge association), or not fluent in Hebrew. The elimination criteria were “very bad” self-reported health or self-reported difficulties in memory and attention abilities. A total of 711 registered members began to fill in the survey; 29 were eliminated due to reports of memory or attention difficulties or very bad health conditions. Of the remaining 682, 524 responded to 100% of the survey questions, but 36 were eliminated because they were not active players. The final sample comprised 488 Israeli Bridge players.

### 2.2. Measures

#### 2.2.1. Demographic Questionnaire

We developed a short demographic questionnaire for this study that included questions related to age, gender, family status, country of birth, employment, education level, residence, and health status.

#### 2.2.2. Players’ Records

The Israeli Bridge Association provided data on six player-level categories and players’ point levels divided into local, national, and international points.

#### 2.2.3. Bridge Motivational Factors Checklist

The Bridge Motivational Factors Checklist (BMFC), developed for this study, is based on qualitative findings published by Judge and Punch [20]. Following focus groups of experts in cognitive rehabilitation and professional Bridge players, the final BMFC version includes eight statements related to possible motivational factors. Five statements address interpersonal factors (e.g., I play Bridge to develop thinking skills); three are social-interaction factors (e.g., I play Bridge to meet other people). Respondents rate each statement on a scale of 1 (do not agree at all) to 5 (mostly agree). A Cronbach’s alpha of 0.73 was found for the total BMFC.

#### 2.2.4. Bridge Cognitive Strategies Questionnaire

The Bridge Cognitive Strategies Questionnaire (BCSQ) was developed for this study to assess the extent to which Bridge players use different cognitive strategies pre-game, in-game, and post-game. The questionnaire was developed based on a cognitive strategies theoretical model [21]. The process to create the BCSQ items followed focus groups that included experts in cognitive rehabilitation and professional Bridge players. The final version includes 21 items of play-related cognitive strategies commonly used pregame, in-game, and postgame. Bridge players examined the BCSQ and confirmed the items and classifications. Respondents rate items according to the extent to which they use each strategy on a 7-point scale from 1 (slightly) to 7 (largely). Cronbach’s alpha was conducted to examine the internal consistency reliability of the total BCSQ and each part. An alpha coefficient of 0.89 indicated high internal consistency reliability of all items. Alpha coefficients of 0.84 for pregame strategies (6 items), 0.85 for in-game strategies (12 items), and 0.76 for postgame strategies (3 items) indicated good internal consistency reliability for the BCSQ.

### 2.3. Procedure

The Ethics Committee of the University of the University of Haifa approved the study (No. 444/19). The Israeli Bridge Association sent the online survey to all its members via email. The questionnaires included informed consent forms. Each participant reported their association ID number and completed the survey.

### 2.4. Data Analysis

Descriptive statistics were performed for sample characteristics, ranges, means, and standard deviations. We performed Pearson correlations to assess relationships between continuous variables and Spearman correlations to assess relationships between ordinal variables. Differences between nominal demographics characterized by player-level categories were analyzed using a Chi-square test. Multivariate analysis of variance (MANOVA) was used to test for differences in the cognitive strategy used in Bridge playing by players of different levels, and univariate analyses of variance (ANOVAs) to examine the source of the significance.

## 3. Results

### 3.1. Sociodemographic and Player Characteristics

**Figure 1:**
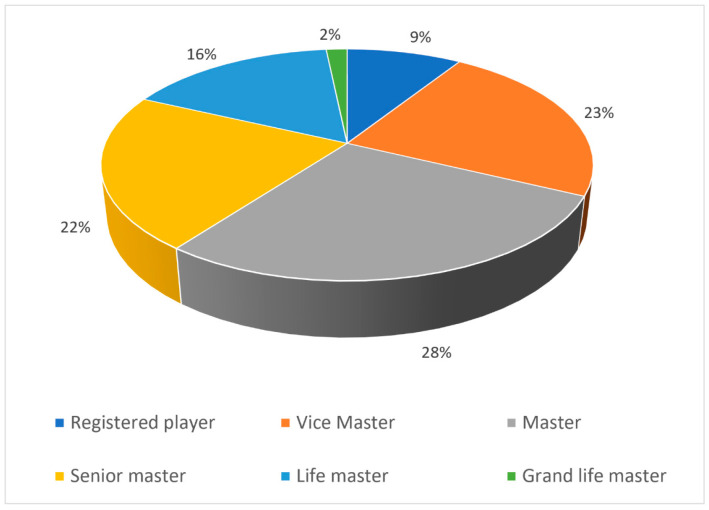
Player level categories.

The respondents’ ages ranged from 19 to 91 years, with a mean age of 68.70 years (*SD* = 9.28). There were 4.5% of respondents aged 19–50 years, 43.5% aged 51–70 years, and the remaining 52% were aged 71 and older. Most (64.5%) respondents were men. The majority were unemployed or retired and reported good general health status. Most (73.0%) players ranged from the Vice Master, Master, and Senior Master categories (Figure 1). Among the players, 75 (15.4%) had gained local points, 224 (45.9%) national points, and 189 (38.7%) international points. Significant negative correlations were found between the players’ age and the number of national (*r* = −0.16, *p* < 0.001) and international (*r* = −0.15, *p* < 0.001) points. Younger players tended to have more national and international points than older players. No significant correlation was found between age and local points (*r* = 0.2, *p* = 0.59). Significant negative correlations were found between age and the player-level category: the higher the age, the lower the player-level category (*r* = −0.2, *p* < 0.001).

Significant differences were found between gender and player-level category, χ^2^ (5, *N* = 488) = 28.0, *p* < 0.001, showing that the men were in higher categories than the women. In addition, employed players were found to be in higher categories, χ^2^ (5, *N* = 488) = 48.73, *p* < 0.001, than unemployed players. No significant player differences were found between player-level categories and the remaining demographic characteristics (Table 1). Additional correlations between sociodemographic characteristics and player records are presented in Supplemental Material (Table S1).

### 3.2. Players’ Motivational Items

Table 2 presents the mean scores of the eight motivation items on a five-point scale. The highest mean was in the item, “I heard it is a fun game” (*M* = 4.20, *SD* = 1.04), and the lowest mean for the item, “I want to play with family members” (*M* = 1.81, *SD* = 1.25), and the total mean score was 3.09 and standard deviation 0.72.

No significant relationships were found between the motivational mean score and player categories (*r* = −0.007, *p* = 0.88) among all players. Additional correlations between sociodemographic characteristics and motivational mean score are presented in Supplemental Material (Table S1).

### 3.3. Players’ Cognitive Strategies

**Figure 2:**
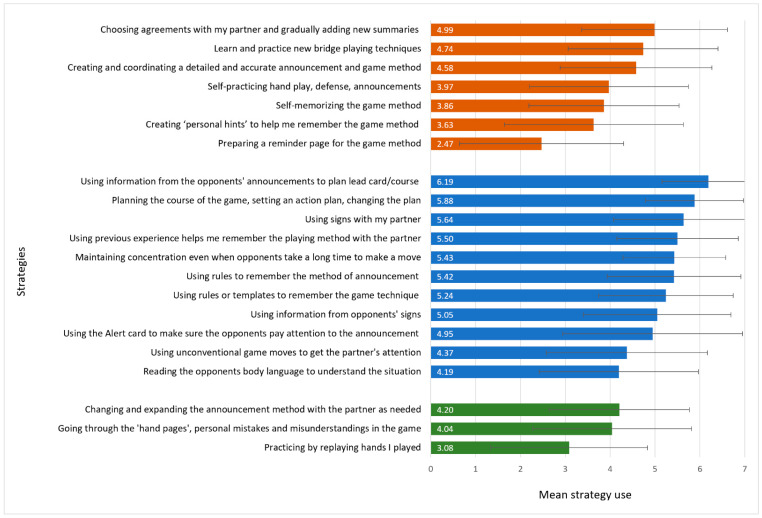
Mean levels of pregame (orange), in-game (blue), and postgame (green) strategy use according to the Bridge Cognitive Strategy Questionnaire.

Analysis of the cognitive strategies players used revealed that the pregame strategy with the highest mean (*M* = 4.99, *SD* = 1.62) was “choosing agreements with my partner and gradually adding new summaries throughout the game”, whereas “preparing a reminder page for the game method” had the lowest mean (*M* = 2.47, *SD* = 1.83). For in-game strategies, “using information from the opponents’ announcements to plan the lead card and the course of the game” was the strategy with the highest mean (*M* = 6.19, *SD* = 1.04), and “reading the opponent’s body language to understand the situation” had the lowest (*M* = 4.19, *SD* = 1.77). Among postgame strategies, “changing and expanding the announcement method with the partner as needed” had the highest mean (*M* = 4.20, *SD* = 1.56), whereas “practicing by replaying hands I played” had the lowest (*M* = 3.08, *SD* = 1.75). Figure 2 presents the mean score of each BCSQ strategy used pregame (orange bars), in-game (blue bars), and postgame (green bars). The items are arranged in each stage from the strategy with the highest to the lowest mean score.

The mean score of all cognitive strategies used by the Bridge players was 4.54 (*SD* = 0.79). Table 3 presents the means and standard deviations of the BCSQ pregame, in-game, and postgame by player level.

MANOVA results yielded a significant difference in strategy use between the players of different levels, *F*(1,5) = 12.02, *p* < 0.001, η_p_^2^ = 0.12. The following ANOVAs found significant differences in the strategy used in-game, *F*(1,5) = 30.99, *p* < 0.001, η_p_^2^ = 0.25, and postgame, *F*(1,5) = 5.70, *p* < 0.001, η_p_^2^ = 0.06, meaning the higher the player level, the more strategies are used. No significant differences between players at different levels were found for strategy use pregame, *F*(1,5) = 0.33, *p* = 0.89, η_p_^2^ = 0.004. Correlations between sociodemographic characteristics and mean cognitive strategies used are presented in Supplemental Material (Table S1).

## 4. Discussion

This study’s main goals and contributions were to examine the characteristics of Bridge players in Israel in terms of demographic characteristics, player records, players’ motivations, and use of cognitive strategies at different stages of the game. The results indicate that most Bridge players registered with the Israeli Bridge Association are self-reportedly healthy, approximately 70-year-old men between the Vice Master and Senior Master categories. These results align with the retirement age in Israel of 67 years. Retirement entails a change in daily routines and creates the need to find alternative activities that compensate for the loss of social relationships with co-workers [22]. Only eight players in the sample were Grand-Master-level Bridge players. Results showed significant associations between younger age, being employed, and higher player-level categories. This means that younger people who are still active in the workforce achieve higher player rankings. The fact that full-time professional and amateur Bridge players can play together even at top-level tournaments allows varying levels of game commitment linked to different ages, motivations, and stages in life among amateurs and professionals [12].

Gender differences in sports and leisure are longstanding and historic in both physical and mind sports, with preference given to men. Even when physical factors are removed, and the sport and leisure activity does not require physical strength as in Bridge, these gender-based divisions continue [23]. Correspondingly, we found in our sample significantly more men than women players and that men reach higher player categories than women.

The results indicate that the study participants are highly motivated to play Bridge, especially because they heard it is a fun game. Enjoyment is important among older adults, who composed most of the sample, and the opportunity to regularly socially connect and have fun with others is an important factor in their choice of activities [24]. In general, the motivation to play Bridge can be supported by sport psychology research addressing many techniques to increase and sustain motivation (strategies to enhance agency beliefs, self-regulation, goal-setting, and others).

The self-determination theory [25] is a common theory that tries to explain motivation in sports [26]. A central element of self-determination theory is the concept of three basic psychological needs: autonomy, competence, and relatedness. The need for autonomy refers to the perception that a person is the “origin” of their actions [27]. The need for competence is associated with experiencing mastery and efficiency in a given environment and social context [28]. The need for relatedness is linked to the perception of experiencing meaningful interactions with significant others in a given context [27]. When all three needs are satisfied within an activity, individuals feel a high degree of autonomous and self-determined motivation. Those three needs reflect the Bridge motivation results of this study. The need for autonomy reflects in items such as “fun game that enhances my mood and health”. The need for competence reflects in items such as, “I want to challenge my brain, and I feel it reduces stress”, and the need for relatedness reflects in items such as, “I want to meet other people, and I want social interaction”. In this study, no significant associations were found between motivation and player records. This can be explained by the composition of the sample, which includes 73% mid-range players (Vice Master, Master, and Senior Master). The limited number of higher-level players did not provide a large enough sample to represent the motivation of the Israel elite players.

Bridge is a complicated game during which many complex situations that entail problem-solving can arise, requiring the use of cognitive strategies [29]. In Bridge, back-and-forth reading of the opponents and the game situation is crucial to strategic interaction. In such strategic interaction, people engage in a series of calculated “moves” [30] designed to further their goals through deception and counter-deception. Through the lens of Bridge as a mind sport, these strategic interactions are intertwined, ongoing performative acts rather than static, achieved, or always successfully performed [30].

The use of different strategies in Bridge, as reflected in Punch et al.’s [4] work, supports this study’s findings that players use most strategies during the game. Similar findings were noted in the current study. These results are expected because, in Bridge, players routinely make mistakes at the table and must operate with incomplete information in high-pressure environments. Furthermore, this study’s results reveal no significant differences in pregame strategy use among the levels of players. Interestingly, the mean score of the cognitive strategy use pregame among the highest level players was lower than the mean score of players of other levels. A possible explanation could be the high-level players’ proficiency and extensive game experience. This proficiency may allow them to invest relatively less effort in strategy use pregame and relatively more in-game and postgame when analyzing and debriefing the course of the game.

Interpreting this study’s results should be considered cautiously. The participants in this sample were all members of the Israel Bridge Association. Therefore, these results may not be generalized to other Bridge players. Because research in the field is scarce, researchers must establish more tools to examine the motivation to play Bridge and the various strategies with which Bridge players of all levels play.

## 5. Conclusions

This study is the first among Israeli Bridge players and one of the first that includes a range of ages and player levels and does not focus on people with determined health diagnoses or specifically on elite players. The results suggest that although Bridge is played by various ages, the active players were mainly above the age of 70 years. Although the total male: female ratio was 3:1, only one woman but seven men had achieved the highest level (Grand Life Master). The current research displays that Bridge is mainly a source of fun, and all levels of players use a wide range of cognitive strategies pre-game, in-game, and post-game. Because Bridge is a strategy game that can be played from childhood to older adulthood, it is important to continue research to further understand the nature and different aspects of the cognitive strategy used among Bridge players and in other mind sports.

## Figures and Tables

**Table 1:** Player-level category by demographic characteristic.

Characteristic	Total	Registered Player	Vice Master	Master	Senior Master	Life Master	Grand Life Master		
	*n* (%)	*n* (%)	*n* (%)	*n* (%)	*n* (%)	*n* (%)	*n* (%)	*χ*2	*p*
Gender								28.000	0.000
Men	315 (64.5)	16 (3.3)	71 (14.5)	82 (16.8)	76 (15.6)	63 (12.9)	7 (1.4)		
Women	173 (35.5)	28 (5.7)	42 (8.6)	55 (11.3)	30 (6.1)	17 (3.5)	1 (0.2)		
Family status									
Married	374 (76.5)	37 (7.6)	85 (17.4)	105 (21.5)	80 (16.4)	62 (12.5)	6 (1.2)	13.950	0.833
Not married	173 (35.5)	7 (1.4)	28 (5.7)	32 (6.6)	26 (5.3)	19 (3.9)	2 (0.4)		
Country of birth									
Israel	278 (57.0)	23 (4.7)	58 (11.9)	75 (15.4)	66 (13.5)	48 (9.8)	8 (1.6)	9.690	0.080
Other	210 (43.0)	21 (4.3)	55 (11.3)	62 (12.7)	40 (8.2)	32 (6.6)	0 (0.0)		
Education level								1.653	0.895
Academic	392 (80.3)	35 (7.2)	93 (19.1)	109 (22.3)	87 (17.8)	61 (12.5)	7 (1.4)		
Non-academic	96 (19.7)	9 (1.8)	20 (4.1)	28 (5.7)	19 (3.9)	19 (3.9)	1 (0.2)		
Employment								48.732	0.000
Yes	176 (36.1)	6 (1.2)	26 (5.3)	42 (8.6)	48 (9.8)	48 (9.8)	6 (1.2)		
No	312 (63.9)	38 (7.8)	87 (17.8)	95 (19.5)	58 (11.9)	32 (6.6)	2 (0.4)		
Health								7.721	0.656
Good	350 (71.7)	32 (6.6)	77 (15.8)	96 (19.7)	82 (16.8)	59 (12.1)	4 (0.8)		
Moderate	113 (23.2)	11 (2.3)	28 (5.7)	33 (6.8)	19 (3.9)	18 (3.7)	4 (0.8)		
Not good	25 (5.10)	1 (0.2)	8 (1.6)	8 (1.6)	5 (1.0)	3 (0.6)	0 (0.0)		

**Table 2:** Ranges, means and standard deviations of the bridge motivational factors checklist.

Item		Range	*M*	*SD*
*I play Bridge because ...*				
1.	I want to challenge my brain	1–5	3.14	1.46
2.	I heard it is a fun game	1–5	4.20	1.04
3.	I want to meet other people	1–5	2.65	1.26
4.	I want to play with family members	1–5	1.81	1.25
5.	I want social interactions with other Bridge players	1–5	3.00	1.26
6.	I feel that it reduces stress in my daily life	1–5	3.08	1.28
7.	It enhances my health	1–5	2.86	1.29
8.	It enhances my mood	1–5	3.98	0.96

**Table 3:** Means and standard deviations of the Bridge Cognitive Strategies Questionnaire.

Player Level	Pregame	In-Game	Postgame
Registered player (*n* = 44)	4.07 (1.2)	4.48 (0.9)	3.28 (1.4)
Vice master (*n* = 113)	3.95 (1.1)	4.75 (1.0)	3.41 (1.3)
Master (*n* = 137)	4.05 (1.2)	5.23 (0.7)	3.72 (1.4)
Senior master (*n* = 106)	4.01 (1.3)	5.57 (0.9)	4.02 (1.3)
Life master (*n* = 80)	4.15 (1.4)	5.95 (0.6)	4.30 (1.3)
Grand life master (*n* = 8)	3.73 (1.2)	6.12 (0.5)	4.04 (0.7)

## Data Availability

The datasets generated and/or analyzed during the current study are not publicly available due to ethical restrictions but are available from the corresponding author upon reasonable request.

## Supplementary Materials

The following supporting information can be downloaded at: https://www.mdpi.com/article/10.3390/ijerph20064968/s1, Table S1: Correlations between sociodemographic characteristics and player points, mean player motivation score, and mean player strategy use.
