# Supplementary material for: Mind Sports: Exploring Motivation and Use of Cognitive Strategies in Bridge
Source: Int J Environ Res Public Health. 2023 Mar 11;20(6):4968. doi: 10.3390/ijerph20064968 (PMC10049287; doi:10.3390/ijerph20064968)
Supplement: Supplementary file 1 [file ijerph-20-04968-s001.zip › ijerph-2169524-supplementary.pdf]

Table S1. Correlations between sociodemographic characteristics and player points, mean player motivation score, and mean player strategy use.

Characteristic		Player points		Player motivation		Player strategy Use	
		r	*p*	*r*	*p*	*r*	*p*
Age		-0.20	<0.001	0.007	0.882	-0.092	0.05
	n (%)	$\chi 2$	*p*	$\chi 2$	*p*	$\chi 2$	*p*
Gender		16.07	<0.001	23.19	0.807	56.11	0.546
Men	315 (64.5)						
Women	173 (35.5)						
Family status		1.83	0.400	43.36	0.054	58.14	0.470
Married	374 (76.6)						
Not married	114 (23.4)						
Country of birth		2.475	0.290	38.00	0.150	51.37	0.718
Israel	278 (57)						
Other	210 (43)						
Education level		1.286	0.526	37.43	0.17	61.93	0.338
Academic	392 (80.3)						
Non-academic	96 (19.7)						
Employment		51.15	<0.001	45.49	0.035	57.67	0.487
Yes	176 (36.1)						
No	312 (63.9)						
Health		2.23	0.693	56.88	0.591	87.20	0.979
Good	350 (71.7)						
Moderate	113 (23.2)						
Not good	25 (5.1)						
